# Reflection on the Integration of Artificial Intelligence in Anaesthesiology: Beyond Algorithmic Performance

**DOI:** 10.4274/TJAR.2026.262383

**Published:** 2026-04-15

**Authors:** Hamza Najout, Mustapha Bensghir

**Affiliations:** 1 Mohammed V University Mohammed V Military Teaching Hospital, Faculty of Medicine and Pharmacy, Department of Anaesthesiology and Intensive Care, Rabat, Morocco

**Keywords:** Airway management, intensive care, perioperative care, pharmacology, regional anaesthesia

Dear Editor,

We read with great interest the review by Dost et al.¹, “Artificial Intelligence in Anaesthesiology: Current Applications, Challenges, and Future Directions.” The authors provide a comprehensive and forward-looking synthesis of how artificial intelligence is reshaping the perioperative continuum, from preoperative assessment to intensive care, education, and research. In particular, their discussion of decision-support systems and predictive analytics raises important questions about how algorithmic outputs should be interpreted and integrated into routine anaesthetic care. Their conclusions are consistent with recent literature describing the rapid expansion of data-driven technologies in modern anaesthetic practice.^2^

While the technical progress described is impressive, several unresolved issues warrant closer attention if innovation is to genuinely improve patient safety. A central concern remains the gap between algorithmic performance and true bedside utility. Many tools achieve excellent technical metrics under controlled conditions, yet their translation into meaningful patient-centred outcomes is far less certain, as highlighted by recent methodological evaluations.^3^ This distinction, clearly acknowledged by Dost et al.^1^, formed the primary motivation for our correspondence.

This tension is particularly evident in the discussion of the hypotension prediction index, which Dost et al.^1^ cite as a prominent example of predictive analytics in perioperative medicine. Although this technology demonstrates strong discriminative ability, its real-world clinical interpretation remains complex. The validation study by Davies et al.^3^ illustrates important methodological considerations in assessing such tools but does not report a definitive positive predictive value in the main text, nor does it demonstrate that false alerts resulted in clinically inappropriate fluid or vasopressor administration. Nonetheless, in theory, frequent alerts with limited immediate clinical relevance may contribute to alarm fatigue or increased cognitive load, a phenomenon well described in the broader literature on physiologic monitoring and patient safety,^4^ underscoring the need for cautious, context-aware integration rather than automated action based solely on algorithmic signals. These considerations should therefore be interpreted as hypothesis-generating rather than as evidence of demonstrated clinical harm or inappropriate intervention.

Equally important are the educational and professional implications of this technological transition. If automated systems routinely identify sonoanatomical structures in regional anaesthesia or anticipate haemodynamic events, there is a legitimate concern that over-reliance could erode independent clinical reasoning. In the absence of robust outcome focused data, these considerations remain perspective-based reflections; however, they are consistent with the well-described concept of automation bias, whereby users may defer excessively to automated recommendations at the expense of independent judgement.^5, 6 ^ To mitigate this risk, artificial intelligence systems should be deliberately positioned as adjunctive tools that support supervised learning, promote reflective decision-making, and preserve the acquisition of foundational skills, rather than replacing core clinical judgement.^2^

Beyond individual tools, Dost et al.^1^ appropriately highlight broader challenges related to governance and responsibility. Medico-legal accountability remains insufficiently defined when clinical decisions are influenced by algorithmic recommendations. Clear attribution of responsibility among clinicians, institutions, and technology providers is essential to ensure that patient safety and professional liability are not diluted as decision-support systems become more prevalent.

To move beyond descriptive concerns and facilitate safe clinical adoption, we propose a pragmatic, stepwise framework for integrating predictive and assistive algorithms into anaesthetic practice: contextual validation, ensuring local performance assessment in the target patient population; human-in-the-loop decision-making, whereby algorithmic outputs inform but do not dictate clinical actions; educational integration, using these tools explicitly as teaching aids within structured supervision; and shared accountability structures, clarifying medico-legal responsibility and documentation when algorithmic advice contributes to clinical decisions. Such an approach may help bridge the gap between technical capability and meaningful clinical benefit.

Concerns regarding equity and generalisability further complicate adoption. Most current models are trained on datasets derived predominantly from high-income settings. When applied to patients with distinct physiological profiles or in resource-limited systems, performance may degrade, risking the emergence of a two-tier standard of care. Recent reviews emphasise that such imbalance may limit the universal value of these technologies unless broader and more diverse validation strategies are adopted.^7^ Importantly, these limitations should not be extrapolated indiscriminately to all forms of artificial intelligence in anaesthesiology; rather, the hypotension prediction index serves as an illustrative, context-specific example within a wider, heterogeneous field. Accordingly, the concerns discussed here should not be extrapolated to artificial intelligence in anaesthesiology as a whole, but rather be understood within the specific context of predictive decision-support tools.

The intent of this correspondence is not to challenge the conclusions of Dost et al.^1^, but to complement their review by proposing a practical framework to support the safe, educational, and accountable clinical integration of such technologies. The next phase of research should prioritise clinical trials focused on patient-centred outcomes rather than surrogate technical metrics, alongside robust external validation, educational safeguards, and clearly defined accountability pathways. Only through such balanced and critically informed integration can technological innovation fulfil its promise as a genuine partner in safe, equitable anaesthetic care.
